# Polycystic Ovary Syndrome: A Disorder of Reproductive Age, Its Pathogenesis, and a Discussion on the Emerging Role of Herbal Remedies

**DOI:** 10.3389/fphar.2022.874914

**Published:** 2022-07-18

**Authors:** Ling-Hui Zeng, Saba Rana, Liaqat Hussain, Muhammad Asif, Malik Hassan Mehmood, Imran Imran, Anam Younas, Amina Mahdy, Fakhria A. Al-Joufi, Shaymaa Najm Abed

**Affiliations:** ^1^ Department of Pharmacology, Zhejiang University City College, Hangzhou, China; ^2^ Department of Pharmacology, Faculty of Pharmaceutical Sciences, Government College University Faisalabad, Faisalabad, Pakistan; ^3^ Department of Pharmacology, Faculty of Pharmacy, Islamia University Bahawalpur, Bahawalpur, Pakistan; ^4^ Department of Pharmacology, Faculty of Pharmacy, Bahauddin Zakariya University, Multan, Pakistan; ^5^ Medical Pharmacology Department, International School of Medicine, Istanbul Medipol University, Istanbul, Turkey; ^6^ Department of Pharmacology, College of Pharmacy, Jouf University, Aljouf, Saudi Arabia; ^7^ Nursing Department, College of Applied Medical Sciences, Jouf University, Sakaka, Saudi Arabia

**Keywords:** PCOS, endocrine abnormality, metabolic disorder, infertility, reproduction

## Abstract

Polycystic ovary syndrome (PCOS) is a very common, complex, and heterogeneous endocrine disorder of women that involves a combination of environmental and genetic factors. PCOS affects women of growing age particularly at the early to late reproductive stage (15–35 years). Currently, PCOS affects 1 in every 10 women worldwide. It is characterized majorly by a raised level of androgens such as testosterone and a large number of ovarian cysts (more than 10) that cause anovulation, infertility, and irregular menstrual cycle. PCOS is also related to other endocrine and metabolic abnormalities, such as obesity, hirsutism, acne, diabetes, insulin resistance, and glucose impairment. PCOS can be treated with allopathic, ayurvedic, and natural or herbal medications along with lifestyle modifications. Herbal medicines remained in demand for numerous reasons such as high cost and side effects associated with the use of allopathic medicine and our traditional norms, which have helped humans to use more herbal products for their health benefits. Estrogenic and nonestrogenic phytochemicals present in various plant species such as *Glycyrrhiza glabra* L. [Fabaceae], *Aloe vera* (L.) Burm. f. [Asphodelaceae], *Silybum marianum* (L.). Gaertn. [Asteraceae], *Serenoa repens* (W.Bartram) Small [Arecaceae], *Actaea racemosa* L. [Ranunculaceae], and *Angelica sinensis* (Oliv.) Diels [*Apiaceae*] are effective and harmless. Herbal medicines are found to be cost-effective, efficacious, and a highly esteemed source of management/treatment for PCOS than allopathic medicines. In this literature review, diagnosis, signs, and symptoms of PCOS; causes of hormonal imbalance; and risk factors associated with PCOS and their management are discussed briefly, and the focus was to find out the role of herbal remedies in PCOS management.

## 1 Introduction

The term PCOS was first used in 1935 by Stein and Leventhal, also named Stein–Leventhal syndrome after the investigators (Knochenhauer et al., 1998; Nagarathna et al., 2014). It is an extremely common and highly prevalent disorder. It affects women of early to late reproductive stage worldwide, but its prevalence is variable among different races and ethnic groups as it is highly prevalent among South Asians than among Caucasians. The occurrence of PCOS is higher among Asian women (52%) than among Western Caucasian women (20%–25%). According to the World Health Organization (WHO), PCOS affects 116 million women (4%–12%) globally in 2012, and in 2020, its ratio increased abruptly to 26% (Carmina et al., 1992; Nagarathna et al., 2014). In Pakistan, 1 in every 10 women is diagnosed with PCOS and this is an alarming situation. In PCOS, follicle-stimulating hormone (FSH) level decreases, and this prevents the maturation of follicles in the final stage. The decreased level of FSH and the increased level of LH and androgens (testosterone) worsen the steroidogenesis. This hormonal imbalance disturbed the normal menstrual cycle of women which makes it difficult for them to get pregnant (Knochenhauer et al., 1998). Among the various causes of infertility, PCOS is considered the commonest and a major cause, accounting for 35%–50% of overall infertility (Carmina et al., 1992). PCOS is not a disease; rather, it is a disorder causing the female ovaries to become enlarged with a large number of cysts (more than 10). These cysts are undeveloped follicles. As the disorder progresses, thickening of the ovary wall occurs, which prevents the release of the ripened follicles known as anovulation (Nagarathna et al., 2014). PCOS is characterized by infertility, disturbance in the normal menstrual cycle, and anovulation (Teede et al., 2010). Women with polycystic ovaries reveal the clinical features of PCOS, including menstrual cycle disturbances, obesity, hirsutism, acne (Archer and Chang, 2004), and abnormality of biochemical profiles, such as elevated serum concentrations of LH, testosterone, androstenedione, and insulin. Insulin resistance is another important factor that contributes to the pathophysiology of PCOS. However, acne, unexpected increase in weight, and increased growth of hair are also clinical manifestations of PCOS (GOLDZIEHER and Green, 1962). Potential factors involved in the pathogenesis of PCOS are generally alterations in neuroendocrine function, steroidogenesis, ovarian folliculogenesis, metabolism, insulin secretion, insulin resistance, adipose cell function, and inflammatory factors, which contribute to the pathogenesis of this disorder. The presence of PCOS is also associated with some cancers, e.g., ovarian cancer and cervical cancer. PCOS is also categorized as endocrine dysfunction in humans because as the disorder develops, it leads to type II diabetes, obesity, ovarian cancer, dysfunctional uterine bleeding, high level of cholesterol, and cardiovascular abnormalities (Dunaif et al., 1989). Moreover, in PCOS, there is imbalance of all the hormones, such as gonadotropin-releasing hormone (GnRH), insulin, the luteinizing/follicle-stimulating hormone (LH/FSH) ratio, androgens, estrogens, growth hormones (GHs), cortisol, parathyroid hormone (PTH), and calcitonin, and all of these hormones are involved in bone metabolism and their imbalance may enhance osteoporosis. Thus, this syndrome also has a relevance with the bone function abnormality (Krishnan and Muthusami, 2017). PCOS is mostly diagnosed when females have trouble in getting pregnant. Diagnosis of PCOS generally consists of detailed family history, appropriate laboratory evaluation, and exclusion of other causes of metabolic disturbances. To treat PCOS, several therapeutic approaches have been tried, comprising diet/lifestyle modifications and the use of medicinal agents, such as oral contraceptive pills or antiandrogens. In recent times, management with inositol has proven to be as rational as beneficial in counteracting the endocrine-metabolic abnormalities associated with this syndrome (Minozzi et al., 2011; Kamenov et al., 2015). As PCOS is a familial condition, it is proving impractical to set up the genetic basis for the syndrome without a clear view of the phenotype. Based on the patient’s response to human corticotrophin-releasing hormone (hCRH), PCOS patients are divided into different classes, e.g., some may show a normal response to hCRH, others may have an exaggerated response of ACTH to hCRH, or some patients may have a high basal level of cortisol and a reduced response to hCRH. PCOS is a complex heterogeneous genetic disorder, and dysregulation of androgen synthesis or androgen excess plays a major role in the pathogenesis of PCOS (Kondoh et al., 1999).

## 2 Etiology of PCOS

The factors associated with the etiology of PCOS may be

### 2.1 Environmental Factors

**FIGURE 1:**
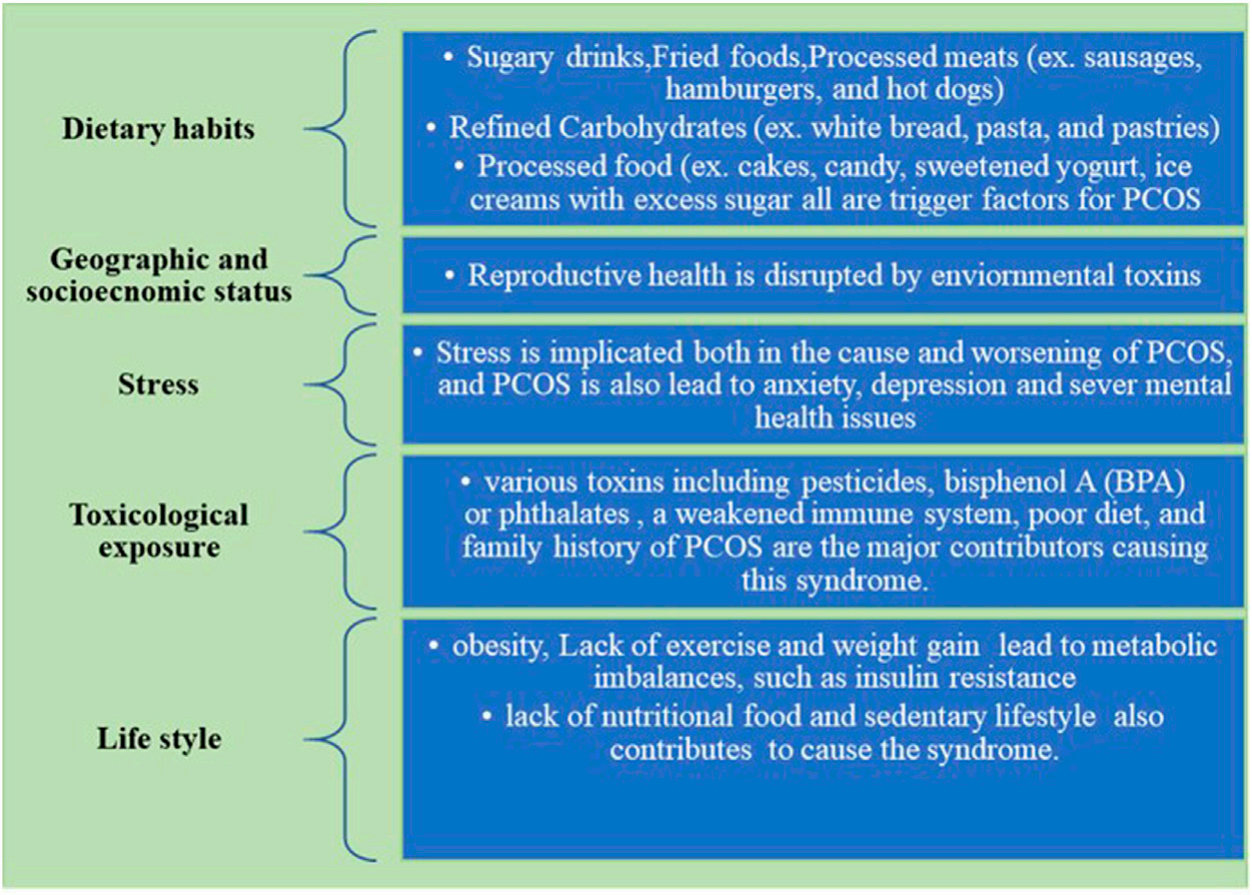
Environmental factors affecting PCOS.

The environmental factors associated with the etiology of PCOS are depicted in detail in Figure 1.

The biggest contributor to PCOS is poor diet and disturbed lifestyle which is also proved in a survey (Lydic and Juturu, 2008). An elevated level of androgens prevents the release of the ovum from follicles. So an unhealthy diet and a stressful lifestyle contribute to the worsening of the symptoms of PCOS.

### 2.2 Genetic Factors

**FIGURE 2:**
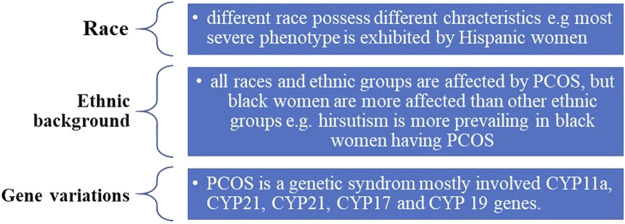
Genetic factors associated with PCOS.

The genetic factors associated with the etiology of PCOS are depicted in Figure 2 **.**

These factors provide an additional insight to determine the epidemiology, prevalence, and presence of PCOS.

Based on these factors, PCOS is divided into four different types.

1) Insulin resistance PCOS: high level of insulin is the common and highly prevalent reason for PCOS (Diamanti-Kandarakis and Christakou, 2009).
2) Adrenal PCOS: stimulation of adrenal secretions during early puberty causes adrenal PCOS; patients with adrenal PCOS generally experience more stress due to excess DHEAS (dehydroepiandrosterone sulfate, an androgen of adrenal glands) (Carmina, 2006).
3) Inflammatory PCOS: chronic low-grade inflammation is generally found in PCOS patients (Duleba and Dokras, 2012).
4) Post pill PCOS: e.g., caused by contraceptive pills and hormonal disturbances (Lara Briden, 2015).

Increased insulin levels and insulin resistance also contribute to the pathogenesis of PCOS.

## 3 Pathogenesis of PCOS

### 3.1 Hormonal Imbalance

**FIGURE 3:**
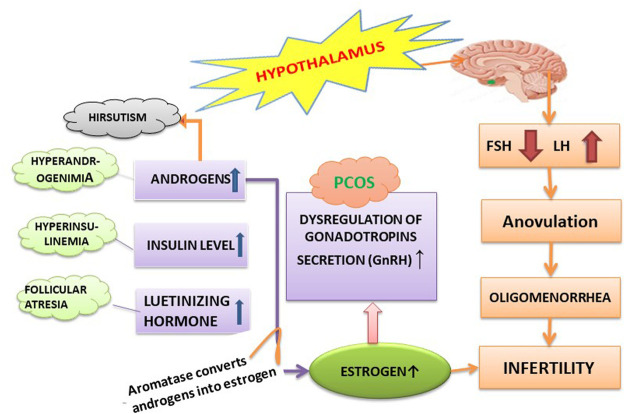
Hormonal imbalance associated with PCOS.

Some major hormones that play a key role in the pathogenesis of PCOS are discussed as follows, as shown in Figure 3.

#### 3.1.1 Androgen

The ovary of an adolescent with PCOS produces androgens excessively (hyperandrogenism), e.g., testosterone, which prevents the maturation of ovarian follicles. So, an immature ovum will be formed that does not release properly, thus leading to anovulation. The ovum and sperm meets for fertilization, and the unavailability of the fully mature ovum is responsible for the conception problem in PCOS patients. A high level of testosterone is also observed in women with PCOS (CHANG et al., 1983; Schneyer et al., 2000; Kumar et al., 2005). However, it is seen in cell function studies of PCOS patients that the androgen response is much exaggerated after its stimulation by exogenous HCG or by endogenous gonadotropin after treatment with exogenous gonadotropin-releasing-hormone (GnRH) analog. It was also observed that the human theca cells culture of PCOS patients produces 20 times more androstenedione than similar cells of normal people (Schneyer et al., 2000).

#### 3.1.2 Insulin

Hyperinsulinemia and insulin resistance are two common contributing factors of anovulation in PCOS patients. Hyperinsulinemia is higher insulin levels in blood, and it mostly happens when production of insulin is higher than its clearance. Androgens also cause insulin resistance, as described in Figure 3 (CHANG et al., 1983). A study conducted in non-obese or less obese PCOS patients suggested that therapy with antiandrogens or androgen suppression improved the insulin sensitivity to a great extent but did not fully restore the insulin sensitivity to normal. Reduction of abdominal adiposity and weight loss in obese patients with PCOS also improved the insulin sensitivity when compared with the weight-matched controlled subjects (Dunaif et al., 1989). The exact causes of metabolic abnormalities remain unclear, but abnormalities in insulin secretion and signaling remain the major cause which was studied in female rhesus monkeys, in which impairments in insulin secretion and action were observed when exposed to androgen excess *in vitro*.

#### 3.1.3 Luteinizing Hormones

It was suggested from different studies that an increased level of insulin is also a contributing factor for anovulation in women with PCOS; it induces premature arrest of follicle development by interacting with LH to augment steroidogenesis. If an unexpected ovulatory cycle occurs and the LH level was monitored regularly for several weeks, it could be seen that the serum LH concentrations suddenly dropped to the standard range (ADASHI et al., 1981). It was also observed in rhesus monkeys and ewes (prenatally androgenized) that the LH secretion remains higher than normal (although significantly lower than in anovulatory subjects). When animals are exposed *in utero* to androgens, a permanent decline in hormonal negative feedback on the hypothalamic-pituitary axis occurs, thus stimulating the androgen’s hypersecretion. The mechanism behind this hypersecretion is not clear; however, recent studies have suggested that in anovulatory patients, the major reason for hypersecretion of LH is irregular negative feedback on LH secretion that is mediated by either estradiol or progesterone (Liu et al., 2012).

## 4 Signs and Symptoms of PCOS

**FIGURE 4:**
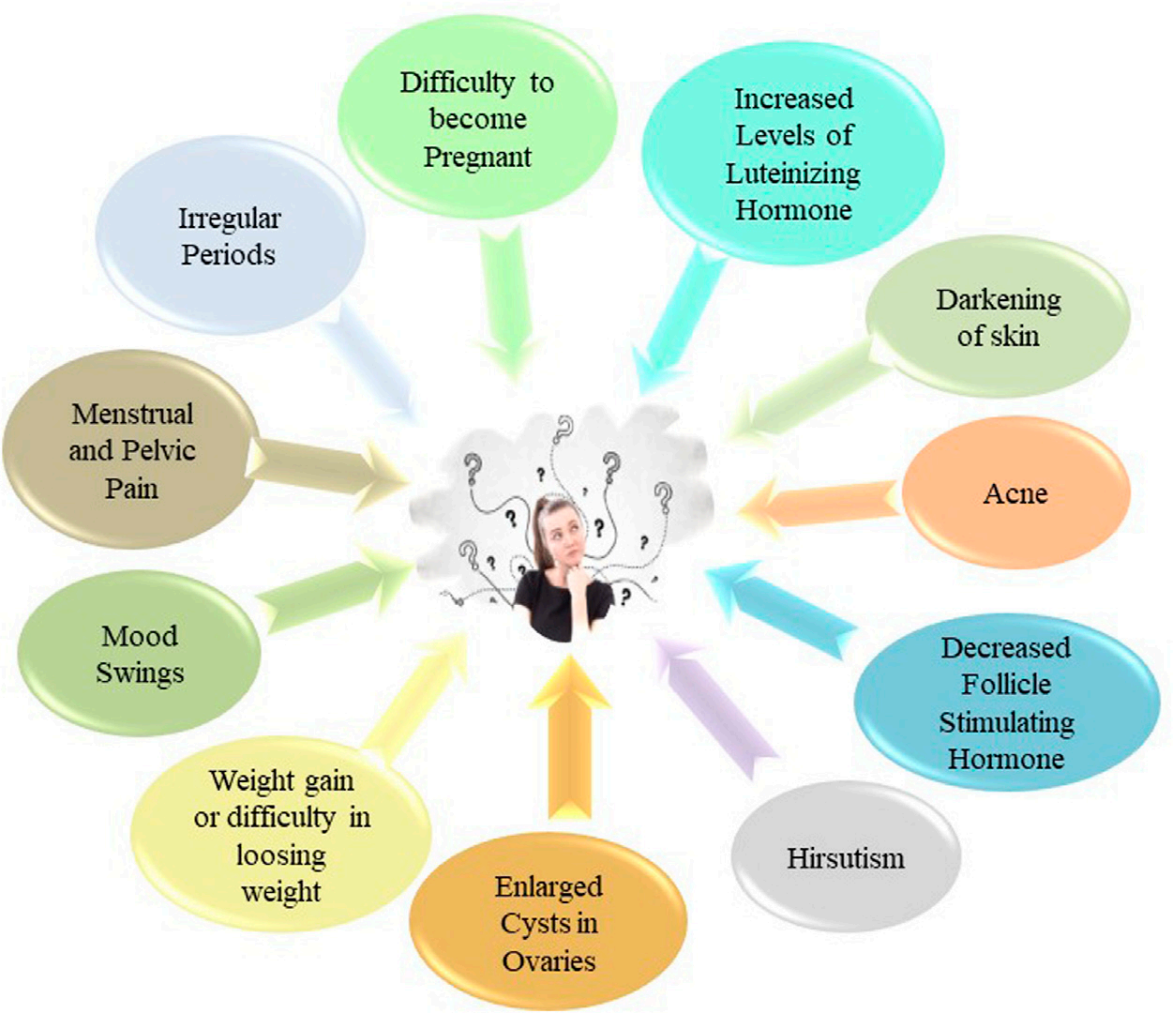
Sign and symptoms of PCOS.

The signs and symptoms of PCOS have a lot of variations and also have interindividual differences. The major symptoms are presented in Figure 4.

## 5 Histopathological Features of PCOS

**FIGURE 5:**
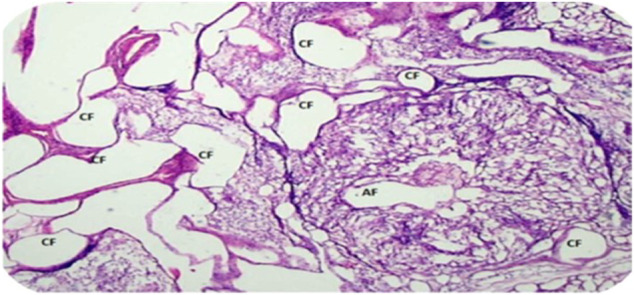
Rotterdam criteria for diagnosis of PCOS and severity of the disorder.

In polycystic ovary syndrome (PCOS), the following changes take place in the ovarian tissues (Abbott et al., 2006): enlarged, sclerotic, and multiple cystic follicles, whole ovarian hypertrophy, thickened capsule >100 μm, increased number of subcapsular follicle cysts, scarcity of corpora lutea or albicantia, hyperplasia and fibrosis of the ovarian stroma, and premature luteinization of theca cells. Figure 5 represents the microscopic view of a female ovary tissue affected with PCOS**.**

## 6 Major Organs Involved in PCOS

The following organs are involved in the pathophysiology of PCOS (Astrup, 2005; Khanage et al., 2019):

1) Ovary: ovary is a female reproductive organ that is present on either side of the uterus and releases the main hormones estrogen and progesterone.
2) Pancreas: it produces insulin in our body.
3) Adrenal gland: this gland is located over both the kidneys and releases an excess of adrenal hormone in patients with PCOS.
4) Pituitary gland: all the hormonal release is controlled by this gland.

## 7 Diagnosis of PCOS

The diagnosis of PCOS is made according to the recommendation of the meeting of the National Institutes of Health Science (NIH) US, which was held in 1990, which states that PCOS should comprise anovulation, hyperandrogenism, or both. However, in 2003, an ESHRE/ASMR joint meeting which was held in Rotterdam suggested that ultrasonography of polycystic ovaries along with hyperandrogenism and anovulation would be sufficient for the diagnosis of PCOS. This was further confirmed in 2006 by The Androgen Excess PCOS Society (AEPCOS). PCOS is mainly a disorder associated with hypogonadism, and secondly, the diagnosis of PCOS is made by either chronic anovulation or ultrasonography of polycystic ovaries (Franks, 2006; Conway et al., 2014; Cappelli et al., 2017).

### 7.1 Criteria for Diagnosis of PCOS

**FIGURE 6:**
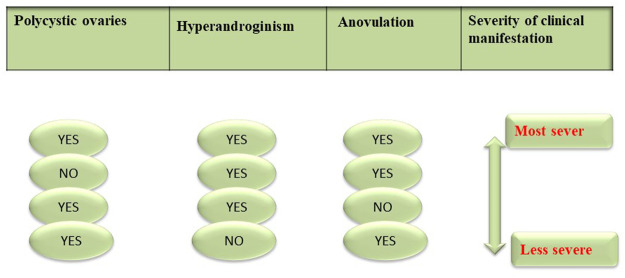
Histopathological features of PCOS ovarian cross section representing changes in the ovary after PCOS. CF, cystic follicles; AF: atretic follicles.

Diagnosis of PCOS is mostly carried out based on the criteria described in Figure 6.

Clinical symptoms of PCOS vary from irregular menstrual cycle or hyperandrogenism to severe metabolic and reproductive disturbances. Currently, pelvic ultrasound is the major tool for the diagnosis of a polycystic ovary. However, Rotterdam criteria as mentioned in Figure 6 are also used when there are multiple symptoms and the condition is worse (Azziz, 2006; Nagarathna et al., 2014). Women with PCOS may not necessarily have a polycystic ovary; similarly women with ovarian cysts may not be diagnosed with PCOS. Clinically, PCOS is diagnosed by:

1. Amenorrhea
2. Sonography
3. Rotterdam criteria (presence of more than two symptoms of PCOS)

If an ultrasound report presents with 12 or more follicles ranging in diameter 2–9 mm or even more in an ovary, then PCOS is confirmed. Even if these follicles are present in one ovary only, it will be sufficient to define PCOS [1].

**FIGURE 7:**
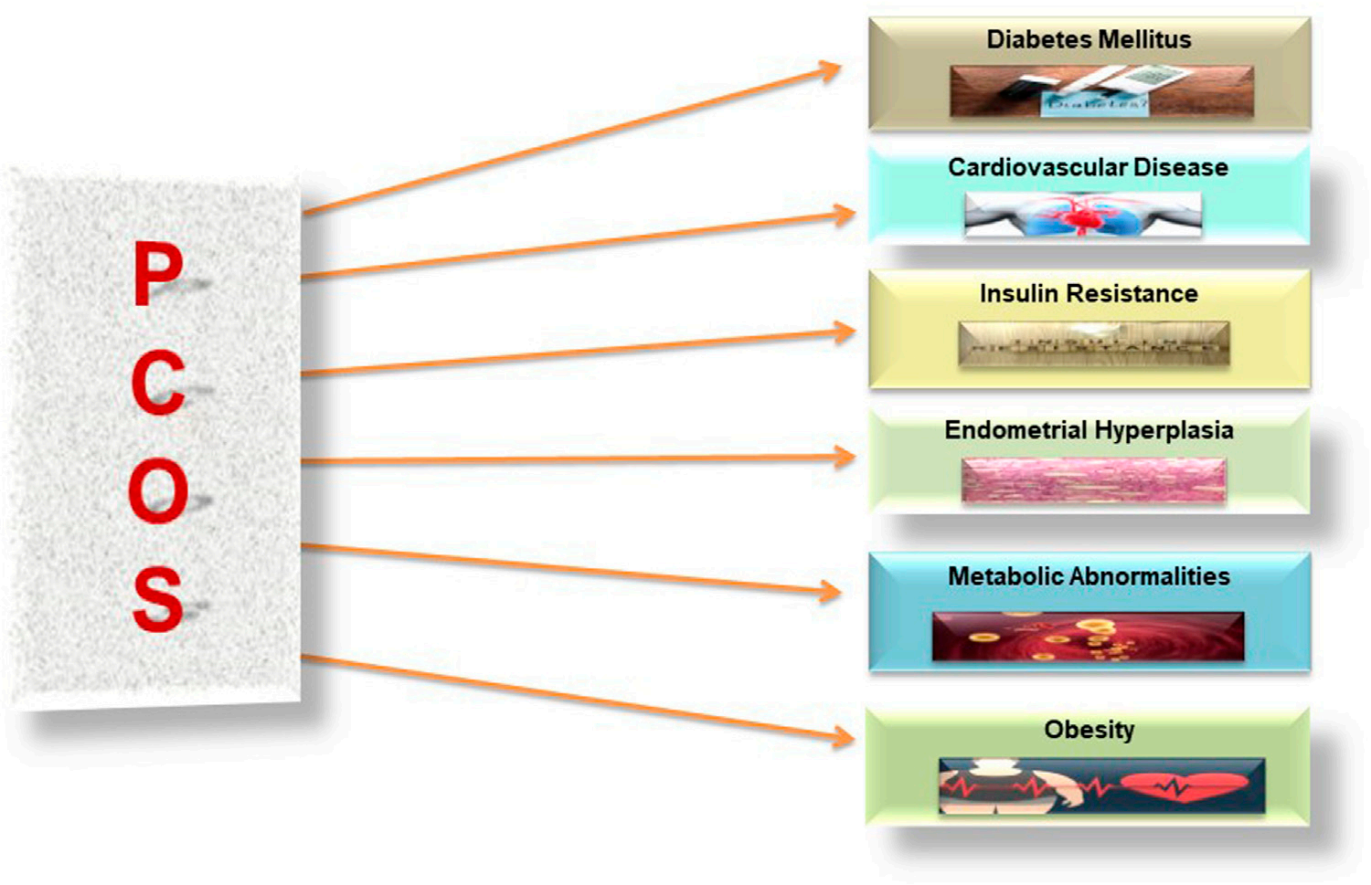
Associated risk factors of PCOS.

Women with PCOS also observed other conditions that contribute to low fertility, e.g., anovulation, increased risks of early miscarriages, and obesity. The risk of developing the following complications increased in PCOS patients as explained in Figure 7 (Shroff et al., 2007).

1. Type II diabetes
2. Cardiovascular disorders
3. Obesity
4. Metabolic syndrome
5. Endometrial carcinoma

## 8 Complications Associated With PCOS

Patients with PCOS are at a high risk of developing some more serious health problems listed as follows in Figure 7.

### 8.1 Diabetes Mellitus and Insulin Resistance in PCOS

It is found that type II diabetes is more prevalent in women with PCOS than in women without PCOS. In most cases, PCOS patients had inherent insulin resistance. Metformin is the most extensively used drug for treating type II diabetes even before insulin secretagogs (Hussain et al., 2014b) and studies suggest that its use throughout pregnancy also reduces the chances of gestational diabetes from 30% to 3% in women with PCOS (Tarkun et al., 2005). In addition to medication, lifestyle improvements have also shown promising results and decreased the risk of diabetes mellitus up to 58% (Conn et al., 2000).

### 8.2 Cardiovascular Diseases in PCOS

The chances of hypertension increases in women with polycystic ovaries. Other complaints in PCOS patients that studies revealed include the following:

• High level of LDL
• Decreased level of HDL
• Disturbance in triglycerides homocysteine
• Type 1 plasminogen activator inhibitor
• Decrease in vascular relaxation
• Disturbance in endothelial function.

Chances of developing coronary artery disease and myocardial infarction also increased more than 7-fold in PCOS patients when compared to the same-age group population without PCOS (Dokras, 2013).

### 8.3 Endometrial Cancer and Endometrial Hyperplasia

Amenorrhea is a major complaint of PCOS patients; if left untreated for a long time, it will develop into endometrial hyperplasia and endometrial cancer. Endometrial hyperplasia is a condition of the female reproductive system. The lining of the uterus becomes unusually thick because of having too many cells (hyperplasia). But in certain women, it increases the risk of endometrial cancer development (Holm et al., 2012).

### 8.4 Metabolic Abnormality

One of the most common metabolic defects in PCOS patients is dyslipidemia. It is reported in multiple studies that the lipid profile of PCOS patients indicates a decrease in HDL (high-density lipoprotein) level and an increase in LDL (low-density lipoprotein) and triglycerides levels (Wild, 2012).

### 8.5 Obesity

Obesity is among the most common metabolic and reproductive abnormalities in patients with PCOS. Clinically, 50% of the cases of PCOS are generally observed to be obese. Lifestyle modifications can reduce obesity because its prevalence is associated with the type of diet intake, less activity, and geographic location (Astrup, 2005).

## 9 Management of PCOS

Polycystic ovary syndrome is not completely curable to date. The treatment used clinically is only to manage the symptoms associated with PCOS (Leena et al., 2016). Hormonal imbalance associated with PCOS is not reversed; improvements in symptoms are mostly associated with lifestyle modifications. Currently, attempts are made to target anovulation, infertility, or management of PCOS-related symptoms.

At present, management options for PCOS are

1. Allopathic therapy
2. Herbal therapy
3. Lifestyle and dietary modifications

### 9.1 Long-Term Management of PCOS (Allopathic Therapy)

Anovulation is accountable for the higher rate of infertility, approximately 75%. It is very difficult for PCOS patients to get pregnant, and if pregnancies occur, chances of miscarriages remain, especially in the first trimester (Homburg, 2004). Anovulation is associated with hormonal imbalance which is an increase in the production of testosterone.

The testosterone level is increased due to (Singh et al., 2010):

• increased LH stimulation,
• hyperinsulinemia; high insulin in blood can impair ovulation and cause the ovaries to make excess testosterone

Level of testosterone hormone can be decreased by the use of the following.

#### 9.1.1 Combined Oral Contraceptives

Oral contraceptives decrease gonadotropin production and thus decrease the testosterone level. The Task Force and the Endocrine Society, the Australian Alliance, and the PCOS Consensus Group recommend combined therapy with hormonal contraceptives for the long-term management of symptoms, such as hyperandrogenism, amenorrhea, or other menstrual irregularities associated with PCOS (Cappelli et al., 2017). These follow the same mechanism comprising suppression of pituitary LH, ovarian androgen secretion, and decrease the testosterone level. Individual oral contraceptives are also effective but at different doses. Antiandrogenic agent, e.g., neutral progestin, is considered effective for symptomatic treatment (Falsetti et al., 2000). Some risk factors, including the elevated blood pressure level, smoking and clotting history, and obesity, associated with the use of oral contraceptives must be considered (Korytkowski et al., 1995).

#### 9.1.2 Clomiphene Citrate

Clomiphene citrate is an antagonist of estrogen receptors, considered as a first-line agent for ovulation induction in PCOS patients. Clomiphene citrate initiates ovulation by stimulating ovarian follicles growth and secretes FSH and LH from the brain’s pituitary gland. After binding to the estrogen receptor, it produces an anti-estrogen effect on the endometrium and cervical mucus. Being economical, with fewer side effects, requiring less monitoring makes it a drug of choice. After the initiation of treatment, pregnancies occur within 6 cycles of ovulation. At high doses, it produces side effects like an increase in the rate of multiple gestations, while in obese patients a high dose is required as they did not respond to therapy (Legro et al., 2007).

#### 9.1.3 Tamoxifen

It works by blocking estrogen receptors in the hypothalamus, which causes ovarian stimulation. Its mechanism of action is similar to CC and hence is used as an alternative to it (Dhaliwal et al., 2011).

#### 9.1.4 Aromatase Inhibitors

These are very potent in inducing ovulation (e.g., anestrazole and letrozole) by stopping the enzyme aromatase in fat tissues (Nugent et al., 2015). Aromatase is necessary for the production of follicles, and it is suggested that in PCOS patients, there is a significant decrease in aromatase level. Decreased estrogen production from the hypothalamus is accountable for increasing the level of gonadotropin-releasing hormone (GnRH) and FSH. Aromatase inhibitors (letrozole) inhibit the conversion of androgens to estrogen by blocking other peripheral pathways, producing positive feedback in the pituitary to increase the FSH level to enhance ovulation (Misso et al., 2012).

#### 9.1.5 Gonadotropins

Exogenous gonadotropins are considered second-line therapy for ovulation in PCOS patients. When treatment with clomiphene fails, then it is the preferred choice to use gonadotropins. These help to induce ovulation, their proper growth, and maturation, so that they are capable of being fertilized. Multiple pregnancies, multiple follicles development, and ovarian hyperstimulation syndrome (hCG-mediated production of vasoactive mediator) are the main drawbacks of gonadotropins use (Nugent et al., 2000).

### 9.2 Drugs for Management of Comorbid Symptoms of PCOS

In PCOS patients, excess androgen level presents symptoms like hirsutism, acne, or alopecia, which may differ among patients.

#### 9.2.1 Metformin

Metformin is an antidiabetic agent used for type II diabetes. It also helps in weight loss and has a lesser effect on lowering testosterone levels. Hirsutism was also improved by metformin (Legro et al., 2007). It improves ovulation and decreases androgen levels. The level of testosterone is generally decreased by weight loss because it will reduce the insulin level by improving insulin sensitivity. Lifestyle modification generally helps in weight reduction.

#### 9.2.2 Troglitazone

It is also an antidiabetic agent found recently to have promising effects on testosterone levels (Dunaif et al., 1996).

#### 9.2.3 Spironolactone (an Aldosterone Antagonist)

Studies suggest that diuretic spironolactone as single-drug therapy reduces hirsutism (up to 40%) by binding to testosterone receptors with general complaints of nausea and menstrual irregularities. Its combination with oral contraceptives further augments the effects up to 75% with a fall in hirsutism up to 45%. It also inhibits adrenal and ovarian steroidogenesis (Cappelli et al., 2017). All these drugs have a promising effect on testosterone, but no drug fully suppresses the testosterone level.

#### 9.2.4 Flutamide

Flutamide belongs to the class nonsteroidal antiandrogens and was found to block the action of both endogenous and exogenous testosterone. For the management of PCOS, combined use of flutamide with metformin shows a synergistic effect; it is also effective against hirsutism. Side effects associated with flutamide, such as dry skin and teratogenicity, limit its use in women with PCOS (Gambineri et al., 2006).

#### 9.2.5 Finasteride

Finasteride is a 5-reductase inhibitor. This enzyme is present in the skin and reproductive tissues. It blocks the production of androgens by inhibiting other forms of this enzyme. By blocking androgens in the hair follicles, it lessens the PCOS-related hair loss (Swiglo et al., 2008).

Teratogenicity is also associated with finasteride use; it has a renowned risk for teratogenicity in male fetuses which can be avoided by adequate contraception.

#### 9.2.6 Statins

In recent studies, statins are also found to be very effective in cardiovascular and endocrine support for PCOS patients (Cassidy-Vu et al., 2016). Experiments showed a reduction in inflammation and improvement in lipid levels and hyperandrogenism. The risk of teratogenicity is also associated with their use in PCOS patients and is still under trial (Unluhizarci et al., 2009).

#### 9.2.7 IVF (*In Vitro* Fertilization) Techniques

IVF is also a choice for patients who were unable to get pregnant by using the single-embryo transfer technique; the risk of multiple gestations can also be overcome. It is observed that the success rate of IVF implantations is the same as that of non-PCOS women because PCOS does not intervene in IVF techniques (Badawy and Elnashar, 2011).

### 9.3 Herbal Therapy for PCOS

The demand for herbal medicines has been increased due to high economic costs and a high number of unfavorable effects associated with the use of allopathic medicines. Since ancient times, herbal plants remain a major source of medicinal preparations (Arentz et al., 2014). Exceptionally, in developing countries, regardless of the great revolution in the pharmaceutical field, the trend of using herbal medicines is increasing day by day (Iqbal et al., 2022). Regardless of the revolution in the field of pharmaceutical chemistry (during the early twentieth century) which makes it easier to synthesize a huge variety of medicinal drug molecules that also allowed the treatment of previously incurable diseases, thousands of medicinal plants that have tremendous action on PCOS symptoms are still in use throughout the world (Badawy and Elnashar, 2011; Arentz et al., 2014; Jazani et al., 2019).

#### 9.3.1 *Glycyrrhiza glabra* L. [Fabaceae]

*Glycyrrhiza glabra* L. a natural herb is used in various medical conditions, such as an expectorant and demulcent, to treat various infections and in osteoarthritis. But it also is found to be very effective in reducing the serum concentration of testosterone and against hirsutism in PCOS patients and is used as an adjuvant in various therapies (Yang et al., 2018). Its effect on androgen metabolism was checked in the luteal stage of the cycle. In this study, nine healthy and young females were incorporated and given a formulation of 7.6% of glycyrrhizic acid only, convectively for two cycles on a daily basis. Plasma and serum tests were performed by using radioimmunoassay techniques. Serum tests include aldosterone, cortisol, and adrenal and gonadal androgen levels while plasma tests are performed to check renin activity. Serum testosterone level decreased within two cycles of treatment (Khanage et al., 2019).

#### 9.3.2 *Aloe vera* (L.) Burm. f. [Asphodelaceae]

The activity of *Aloe vera* (L.) Burm. f. gel against PCOS was checked in a rat model. In this study, five-month-old Charles Foster female rats were included. To induce PCOS, they were fed with an aromatase inhibitor drug letrozole. Treatment of these rats with an oral formulation of *Aloe vera* gel for 45 days showed considerable effectiveness against PCOS. In Charles Foster rats (female rats), it restores the steroid status in ovaries and altered the steroidogenic activity and estrus cyclicity (Maharjan et al., 2010).

#### 9.3.3 *Linum usitatissimum* L. [Linaceae] (Flaxseed)

Flaxseed powder also shows some promising results in PCOS patients by reducing androgen levels and hirsutism (Nowak et al., 2007). This effect was evaluated in a case study and a significant decrease in hirsutism and androgens was observed. In this case study, flaxseed supplementation (at a dose of 30 g/day) was administered to a female patient. The treatment duration was four months; after this period, the effect on her hormones was observed. These four months of follow-up showed a significant improvement in PCOS-related symptoms, especially a decrease in hirsutism, obesity, and insulin and serum concentrations of testosterone.

#### 9.3.4 *Cinnamomum verum* J. Presl. [Lauraceae] (Cinnamon)

Cinnamon extract was found to be very effective in improving IR and potentiating insulin action (Sun et al., 2004). It exerts its main action on the insulin-signaling pathway by increasing P13-K activity and thus reducing insulin resistance. Its effect was checked in a randomized study trial. In this study, 15 women with PCOS were selected and administered with oral cinnamon extracts daily and then with placebo for eight weeks. The results showed a significantly reduced insulin resistance in the drug-treated group while no effect is seen in the placebo group (Qin et al., 2003).

#### 9.3.5 *Curcuma longa* L. [Zingiberaceae] (Curcumin)

Effect of *Curcuma longa* L*.* on the polycystic ovary was observed in a female rat model that showed very favorable results. In the letrozole-induced female Wistar rat, its extracts were administered. The decrease in androgens level and improvements in ovulation were similar and as good as clomiphene citrate (Nabiuni et al., 2015).

#### 9.3.6 *Actaea racemosa* L. [Ranunculaceae] (Black Cohosh)

*Actaea racemosa* L. is used to treat various medical conditions related to hormonal disturbance, e.g., mood swings, anxiety, abdominal cramps related to periods, and menopause (PMS). However, various side effects are associated with its use, including GIT issues, obesity, headache, muscular pain, and vaginal spotting (Fan et al., 2021).

#### 9.3.7 *Paeonia lactiflora* Pall. [Paeoniaceae] (White Peony)

This herb is used to correct the level of the altered hormone that causes PCOS. In PCOS patients, the progesterone level is suppressed, but its use suppresses the LH and regulates the level of progesterone. When used regularly in the form of tea, it regulates estrogen and prolactin secretion. It contains various phytochemicals that help in hormonal regulation (Takahashi and Kitao, 1994; Westphal et al., 2006).

#### 9.3.8 *Vitex agnus-castus* L. [Lamiaceae] (Chaste Berry)

This herb has a very prominent effect on the pituitary gland. As various hormones involved in PCOS pathology are released from the pituitary gland, it also has the potential to treat PCOS symptoms like anovulation, amenorrhea, and pelvic pain. It is one of the most commonly used ancient drugs for hormonal regulation. As this herb affects the hormonal level, its use is prohibited in pregnant women or those taking birth control pills and also in people taking Parkinson’s medications or antipsychotics. In a study, 93 women who had tried to conceive for 1–3 years were given a combined formulation containing chaste tree, arginine, vitamins, and minerals in a dose of 1–4 ml of dried berries daily (1:2 dried plant tincture of 500–1,000 mg) and the effect was observed in the supplementation and placebo groups. After three months, increased mid-luteal progesterone was observed in the supplementation group with normalized menstrual cycles and there were no significant changes in the placebo group. The supplementation group contained 53 women of whom 14 become pregnant, while in the placebo group four of the forty women conceived and three other women conceived after 6 months in the supplementation group (Westphal et al., 2006; Goswami et al., 2012).

#### 9.3.9 *Urtica dioica* L*.* [Urticaceae] (Stinging Nettle)

In patients with polycystic ovary syndrome, the sex hormone-binding globulin (SHBG) level is very low and the production of the male hormone testosterone is more. This plant is used to decrease the testosterone level and increase the production of SHBG, and thus, correct the hormonal imbalance in PCOS patients. The phytochemicals causing the effect are abundantly present in the root of the plant. Long-term use of this plant can cause hypotension (Najafipour et al., 2014; Zare et al., 2015).

#### 9.3.10 *Camellia sinensis* (L.) Kuntze (Theaceae) (Green Tea)

Green tea is a commonly used herbal remedy for weight loss (Ghafurniyan et al., 2015). Green tea modulated the gonadotropin level, reduced IR and rat’s weight, and also improved the ovarian morphology. Moreover, it reduced the ovarian cyst and also improved the ovarian morphology (Ghafurniyan et al., 2015).

#### 9.3.11 *Silybum marianum* (L.) Gaertn. [Asteraceae] (Milk Thistle)

*Silybum marianum* (L.) Gaertn*.* was found to be very effective in hormonal regulation but its combination with metformin proved more beneficial in treating PCOS symptoms, such as anovulation. Mannerås et al. (2010) studied its effects on the levels of glucose, insulin, progesterone, LH, and testosterone in humans. Sixty patients recruited in a study were divided into 3 groups containing 20 individuals. The first group received *Silybum marianum* (L.) Gaertn. only in a dose of 750 mg/day in a divided dose. The second group was given metformin 1500 mg/day only in a divided dose, while the third group was given both *Silybum marianum* (L.) Gaertn*.* and metformin in combination with the same doses as given to the other groups. After three months of follow-up, a significant increase in progesterone level was found. The combination of *Silybum marianum* (L.) Gaertn. and metformin was found to be very effective in managing the symptoms of polycystic ovary syndrome, such as the ovulation rate.

#### 9.3.12 *Gymnema sylvestre* (Retz.) R. Br. ex Sm. [Apocynaceae] (Gymnema)

*Gymnema sylvestre* (Retz.) R. Br. ex Sm. is a traditional herb known due to its antidiabetic and lipid-lowering action. Its antidiabetic activity is probably due to its nutritive restorative action on the *β*-cells of the pancreas. It also regulates insulin activity and reduces the increased triglycerides level related to PCOS that was found to be very effective in eradicating the symptoms of PCOS. Its active constituent is gymnemic acids which is a saponin. If gymnemic acid is taken before a meal, it masks the sweet sensation by suppressing the taste. Hypoglycemic activity of *Gymnema sylvestre* (Retz.) R. Br. ex Sm. has established in various experimental models of diabetes that it regulates blood sugar in hyperglycemic patients at a daily dose of 3.5–11 ml (1:1 liquid extract) (Hywood, 2004; Khanage et al., 2019).

#### 9.3.13 *Trifolium pratense* L. [Fabaceae] (Red Clover)

*Trifolium pratense* L. increases the progesterone level in the body. The phytochemical isoflavones are responsible for their medicinal activity. It also has detoxifying properties that was used for the treatment of acne, an associated symptom of PCOS. Side effects associated with the use of *Trifolium pratense* L. are headaches, vomiting, occasional vaginal bleeding, muscular spasm, and rash. Its use should be avoided in conditions such as endometriosis, breast cancer, and ovarian cancer because it increases the progesterone level in the body which may worsen these conditions. *Trifolium pratense* L. is also prohibited in pregnancy and breastfeeding or if one has any kind of bleeding disorder because it also increases the chances of bleeding (ABBASIAN and GHANBARI, 2017).

#### 9.3.14 *Sesamum indicum* L. [Pedaliaceae] (Sesame Seeds)

The plant part used as medicine is seeds which contain many beneficial nutrients effective in the management of PCOS. Its black seeds reduce testosterone levels, increase insulin absorption, and regulate menstruation. Abundant lignans, phytosterol, vitamins B1, B6, calcium, magnesium, and zinc help in hormonal balance (Goswami et al., 2012; Ghasemzadeh et al., 2013).

#### 9.3.15 *Cucurbita pepo* L. [Cucurbitaceae] (Pumpkin Seeds)

It is found to be very beneficial in eradicating the symptoms associated with polycystic ovary syndrome. It contains omega 3 fatty acids that are useful for hyperinsulinemia and to regulate high cholesterol levels. It is also found to be a very rich source of beta-sitosterol that is involved in the reduction of excess testosterone levels. It is also beneficial in treating other symptoms of PCOS, such as acne, hirsutism, and obesity (Szczuko et al., 2017).

#### 9.3.16 *Oenothera biennis* L. [Onagraceae] (Evening Primrose Oil)

Studies found that the phytoestrogenic chemicals present in the evening primrose oil are very effective as they act on the hypothalamic-pituitary axis. An experimental study was carried out in 30 rats (female Sprague–Dawley rats) with a regular sexual cycle. Evening primrose oil in 1000 mg/kg and 2000 mg/kg doses were used. Results showed that this oil has wonderful effects on lowering luteinizing hormone/FSH and testosterone levels (Meletis and Zabriskie, 2006; Zand Vakili et al., 2018).

#### 9.3.17 *Serenoa repens (W.Bartram)* Small [Arecaceae]

Hirsutism is among one of the major symptoms associated with PCOS that occurs due to the increased production of estrogen hormone. *Serenoa repens* (W.Bartram) Small is a herb that helps to treat hirsutism because of its antiandrogenic activity; in addition, it reduces obesity and increases libido. A combination of *Serenoa repens (W.Bartram)* Small with *Vitex agnus-castus* L. helps to restore hormone balance in PCOS patients as described in Table 1 (Vassiliadi et al., 2009; Nagarathna et al., 2014).

**TABLE 1:** Combined therapies for the management of PCOS.

Serial no	Drug name	Combined effect	Reference
1	Clomiphene citrate + myo-inositol	Improves ovulation induction	Kamenov et al. (2015)
2	Diane35 + *Astragalus caprinus* L.	Improves insulin resistance and ↓ high androgen hormone status	
3	Myo-inositol + oral contraceptive pill	Decreased hyperinsulinemia and effective control on endocrine, metabolic, and clinical outcomes	Minozzi et al. (2011)
4	Combined herbal treatment with *Glycyrrhiza glabra* L*., Cinnamomum verum* J. Presl., *Tribulus terrestris* L., and *Hypericum perforatum* L. with lifestyle modifications	↓Oligomenorrhea, BMI, insulin, and LH levels and ↑Pregnancy rates and quality of life	Speelman (2019)

5	*Commiphora mukul* (Hook. ex Stocks) with clomiphene citrate	Reduce the serum testosterone level	Pandey et al. (2017)
6	Clomiphene citrate + *Cimicifuga racemose* L.	Improves cycle outcomes and pregnancy rates	Shahin and Mohammed (2014)
7	*Serenoa repens* (W.Bartram) Small. (Saw palmetto) + *Vitex agnus-castus* L.	Restore hormonal balance	Vassiliadi et al. (2009)

#### 9.3.18 *Tribulus terrestris* L. [Zygophyllaceae] (Puncture Vine)

*Tribulus terrestris* L. is very effective in treating menstrual irregularities, also effective in increasing ovulation, and also has an antidiabetic effect; so it is a wonderful choice for PCOS patients (Arentz et al., 2014; Parikha and Krishna, 2019).

#### 9.3.19 *Mentha spicata* L. [*Lamiaceae*] (Spearmint Tea)

This herb has some antiandrogenic properties that were observed in a study. The study was conducted in Turkey at two centers as a thirty-day controlled and randomized trial [59]. Forty-two participants were randomly selected, observed, and compared with a placebo group for a month after giving spearmint tea (bid). Hormone levels of serum androgen and gonadotropin were checked at an interval of 15 days. In the spearmint tea group, all the patients (except one) showed a reduction in hirsutism and testosterone levels over the 30 days while the LH and FSH levels were raised (Grant, 2010; Goswami et al., 2012).

#### 9.3.20 *Matricaria chamomilla* L. [*Asteraceae*] (Chamomile)

*Matricaria chamomilla* L. was found to reduce the testosterone level. Thirty Wistar rats (virgin adult cycling) having weights 200–220 g were distributed into 2 groups and housed in a cage under standard conditions (21 ± 2°C, 12-h light/dark cycles) for at least one week before and throughout the study. Vaginal smears were obtained between 8:00 and 12:00 h to check the estrous cyclicity of virgin rats. An intramuscular (IM) injection of estradiol valerate (2 mg in 0.2 ml of corn oil) was given after about 4 days to induce PCOS. To the control group, only corn oil was injected, and after sixty days of treatment, the experimental group was checked for follicular cysts. PCOS-induced rats were administered intraperitoneally by multiple doses (25, 50, 75 mg/kg) of the alcoholic extract of *Matricaria chamomilla* L. for 10 days. The results demonstrate that *Matricaria chamomilla* L. can reduce the total testosterone levels while the LH level was raised. However, no effect was observed on lipid parameters, LH/FSH ratio, and DHEAS level (AMIR et al., 2007).

#### 9.3.21 *Astragalus dasyanthus* Pall. [Fabaceae] (*Astragalus* Polysaccharide)

*Astragalus* polysaccharide aids in the metabolic regulation of PCOS symptoms. It is more beneficial when used in combination with Diane-35. This combination indicated an improvement in insulin resistance, lipid metabolism, and reduction in high testosterone levels. This effect was studied in 35 women with PCOS by giving a combined formulation of *Astragalus* and Diane-35 for 3 months. Lipid profile, hormonal levels, and insulin sensitivity are checked before and after the administration of drugs. At the end of the treatment, results were a decrease in fasting serum insulin and LH/FSH level which support the reduction of PCOS symptoms (LUO et al., 2009; Nagarathna et al., 2014).

#### 9.3.22 *Foeniculum vulgare* Mill. [Apiaceae]

*Foeniculum vulgare* Mill. (fennel) has been traditionally used for the treatment of anovulation/infertility. Fennel has very strong anti-inflammatory, estrogenic, and antioxidant properties due to which it has the potential to treat PCOS. Dr. Karampoor and colleagues evaluated the effects of the hydroalcoholic extract of fennel on female Wistar rats with PCOS. They found that the FSH level was enhanced while the LH and testosterone levels were decreased (Karampoor et al., 2014). Ghavi and colleagues conducted a randomized double-blind, placebo-controlled trial and found that fennel essence was not very effective in abolishing the ovarian cyst symptoms but has some effects on the ovarian follicle and dehydroepiandrosterone sulfate (DHEAS) levels (Ghavi et al., 2019). In another study, researchers loaded the *Foeniculum vulgare* Mill. extract on chitosan-engaged tripolyphosphate ions as a cross-linking molecule and found that this encapsulated formulation was better in controlling the symptoms of PCOS as compared to fennel-alone extract (Bayrami et al., 2020).

#### 9.3.23 *Ferula asafoetida* (Falc.) H.Karst. [Apiaceae]

Fatemeh Ghavi and colleagues found the effects of *Ferula* on androgenic hormone levels and ovarian structure in patients with PCOS. They conducted a triple-blind controlled clinical trial in which 34 students were selected randomly and separated into two groups. The treatment group received 100 mg of *Ferula asafoetida* (oleo-gum resin), while oral paraffin (placebo) was given to the control group twice daily for 3 months. After three months of administration, the efficacy of this herb was evaluated in the treatment groups. The level of DHEAS, TSH, testosterone, and the number of cystic follicles decreased significantly in the treatment groups; however, there was no significant change in the hormone level of FSH, LH, and prolactin (Ghavi et al., 2020).

## 10 Lifestyle Modification

A deskbound lifestyle and a diet of fried foods, processed meats, sausages, hot dogs, and a diet rich in fat and carbohydrates, such as intake of too much sugar and carbonated drinks, cause obesity and insulin and hormonal imbalance that cause PCOS by stimulating androgen receptors present outside the ovary. Lifestyle improvements, such as exercising regularly, taking a healthy diet, and avoiding too much dairy and fast food consumption, help lose weight and improve insulin sensitivity in PCOS patients. So lifestyle improvement should be the first-line regimen for PCOS patients. Unfortunately, all these treatments have only a temporary effect and for permanent weight loss, one has to stick with this routine throughout life. It is observed that in 90%–95% of the cases, the results are not sustained. For sustained weight loss in markedly obese individuals, bariatric surgery is the only treatment option. According to the current recommendations of the National Institutes of Health, bariatric surgery is used in patients having a BMI of more than 40 or less than 35 but with some other serious medical conditions. As women with polycystic ovary syndrome also have other symptoms related to hormonal and endocrine abnormalities, bariatric surgery shows a significant improvement in PCOS symptoms, and it may imply a cure for the polycystic ovary syndrome (Norman et al., 2002; Hoeger, 2006).

### 10.1 Dietary Intake

A diet of low sugar and saturated fat content helps control insulin resistance, cardiovascular abnormalities, and menstrual irregularities in PCOS patients (Lin and Lujan, 2014).

#### 10.1.1 Myo-Inositol

Inositol is a natural molecule found as phosphates in the cell nucleus, phospholipids in cell membranes, and lipoproteins in the plasma. Inositol may promote ovulation in PCOS patients (Unfer et al., 2012; Bevilacqua et al., 2015). Another study carried out in 20 (obese) PCOS patients found that after three months of supplementation with myo-inositol, the menstrual cycle is completely restored in all amenorrheic and oligomenorrheic subjects, and insulin irregularity and reproductive hormone balance are also improved (Kalra et al., 2016). In 2011, experimental research results showed that a combined treatment of myo-inositol and contraceptive pill of four gram of the daily dose is more useful in reducing PCOS symptoms than the contraceptive pill alone, as described in Table 1.

### 10.2 Exercise

Exercise aids in weight loss as was evaluated in a study carried out in an estradiol valerate PCOS rat model. This was to check the impact of exercise (on computer-monitored wheels) on ovarian structure (morphology) and the change in the hallmarks of PCOS. The rats were separated into 4 groups: 1- a control group given oil; 2- an exercise group given oil + exercise; 3- polycystic ovaries induced group by estradiol valerate administration; and 4- an exercise group of PCOS induced by estradiol valerate. Two groups of rats were selected: in one group PCOS was induced by injecting estradiol valerate (IM) and in the control group, only oil was administered. The effect of exercise on ovarian structure, protein expression of nerve growth factor (NGF), mRNA, and several ovarian cells expressing the p75 neurotrophic receptor in rats (EV-induced PCOS) were evaluated. It was observed from the results that ovarian morphology was normalized in the PCOS exercise group; mRNA, NGF, and protein concentrations were also normalized in the PCOS exercise group; and reduction in NGF receptor-expressing cells in polycystic ovaries was observed. Overall, the study showed a favorable effect of regular exercise in the prevention and management of PCOS (Manni et al., 2005).

## 11 Critical Discussion

Polycystic ovary syndrome is a heterogenic disorder with complex and uncertain etiology. PCOS patients show some hallmark features, such as ovarian cysts (ovaries with a collection of fluid), irregular or delayed menstrual periods, weight gain, fatigue, thinning of hair, infertility, acne, pelvic pain, headaches, sleep problems, unwanted hair growth, and mood changes. As the disorder progresses, chances of other risk factors also increased, including diabetes mellitus, high blood pressure, and abnormal lipid profile. If PCOS is diagnosed initially, it can be managed properly with lifestyle modification, and the onset of other complications such as type II diabetes may be delayed or prevented. The purpose of PCOS treatment is to correct or normalize ovarian function. Conventional therapeutic options provide only symptomatic relief from these symptoms; no treatment provides complete cure for the disorder. Currently, therapeutic options for PCOS vary from lifestyle changes to pharmacological treatments. Herbal remedies can be considered as a convenient option for PCOS management because they have fewer side effects than allopathic medications. Most recently, herbal remedies have realized a twirling point. If the benefits of herbal therapies are compared with other available treatment options, then herbal therapy will be preferred due to fewer side effects, cost-effectiveness, and the presence of multiple phytochemicals in a single preparation (Hussain et al., 2014a; Hussain et al., 2015; Asif et al., 2022). Administrations of allopathic medicines, such as metformin, oral contraceptives, and clomiphene citrate, are related to several unwanted side effects including nausea, vomiting, and gastric pain that may necessitate the termination of therapy. Thus, the combined treatment with natural products, such as inositols, lipoic acid, evening primrose oil, milk thistle, and saw palmetto, represents a valid and well-tolerated substitute. Antiandrogenic effect of *Glycyrrhiza glabra* L*.* was also examined in various clinical studies, such as lowering of testosterone levels in the healthy female during menstrual cycles. Moreover, obese women were treated with *Cinnamomum verum* J. Presl. and *Fagonia indica* Burm f (Younas et al., 2022) to alleviate oligo/amenorrhea and other PCOS-related symptoms, and green tea consumption improves the associated symptoms of PCOS, such as obesity, hyperinsulinemia, and hyperandrogenemia. PCOS is a complex heterogeneous metabolic disorder that requires long-term management, and treatment with allopathic drugs of choice can be very costly with a large number of associated adverse effects. Thereby, herbal therapy is useful in the treatment of the implicating factors of PCOS: easy availability to provide relief from symptoms and action as an immune system booster makes it a better choice. The chosen herbal therapy can be aided by combining a PCOS-friendly diet and exercise to attain more promising effects, and a few of these combined treatment/management options are discussed in Table 1.

## 12 Conclusion

Over the past decade, herbal medicine usage by women has increased. In relative studies of allopathy, homeopathy, and ayurveda, allopathic medication does not provide full relief from PCOS but helps in controlling the symptoms; the biggest drawback associated with the use of allopathic medication is that it requires more money and duration. Furthermore, there is a need for more treatment options for this debilitating disorder named polycystic ovary syndrome. All these factors necessitate the development of a combined formulation with reduced cost, duration, and side effects of the prevailing treatments**.** Herbal medicines are chosen for the management of PCOS depending on their collective synergistic effects in clinical trials. Those drugs that have shown to be more efficacious than an individual herbal agent in numerous clinical trials will be chosen preferably, e.g., *Astragalus caprinus* L. and Diane-35 or contraceptive pills and myo-inositol when used in combination proved more efficacious. This literature review is useful in understanding the helpfulness of herbal medicinal plants in the cure of PCOS. In this review, we have discussed all the easily available, cost-effective herbal drugs with a potential effect on alleviating the PCOS symptoms.
